# Shaping the Research Agenda

**DOI:** 10.1371/journal.pntd.0003350

**Published:** 2015-01-08

**Authors:** Edith Certain, Robert F. Terry, Fabio Zicker

**Affiliations:** 1 Former staff of TDR, the Special Programme for Research and Training in Tropical Diseases, Geneva, Switzerland; 2 Special Programme on Research and Training in Tropical Diseases (TDR), a co-sponsored programme of UNICEF/UNDP/World Bank/WHO, and based at the World Health Organization, Geneva, Switzerland; 3 Center for Technological Development in Health (CDTS), Fiocruz, Rio de Janeiro, Brazil; René Rachou Research Center, Fiocruz, Belo Horizonte, Brazil, Brazil

## Introduction

Despite the global efforts in research, training, and control over the years, tropical diseases remain a major cause of ill-health in poor populations. An estimated 2.7 billion people living on less than US$2 per day, in both rural and impoverished urban areas of low-income countries, are affected [1], [2]. Thirteen neglected tropical diseases alone account for 534,000 deaths each year and the loss of 57 million disability-adjusted life years (DALYs) [3].

It has been the poverty of the affected populations that remains one of the biggest challenges to the research and development of new health products reliant largely on a market-based approach [4]. Moreover, apart from their vote at the World Health Assembly, many developing countries had little say in setting research priorities. The prevailing paradigm in research, as in development, was one of donor and recipient [5].

One of the key tenets of the Special Programme for Research and Training in Tropical Diseases (TDR)'s strategy has been to counter this imbalance by focusing on the health priorities from a developing country's perspective and the public health need and giving a voice to these priorities by engaging with the researchers and populations most affected [6].

In this article, we give some examples of how the TDR approach of a wide engagement of stakeholders has helped develop a more equitable research agenda that TDR has used for its own funding decisions, but even more importantly, this approach has helped shape the global research agenda on neglected infectious diseases of poverty by creating new collaborations and attracting greater research and implementation support from international funders and governments.

## Putting Developing Countries in the Driving Seat

TDR's Scientific and Technical Advisory Committee (STAC) was established by engaging leading scientists from around the world to set the Programme's overall research priorities. Over the years, Scientific Working Groups (SWG) were also established to provide updates and peer review for specific areas producing several priority setting reports. This peer review system gave scientists from disease-endemic countries control over TDR's priorities and projects while also giving scientific credibility and organizational flexibility to the WHO. The balance between consultants from disease-endemic countries and from developed countries improved with the increased availability of well-trained and experienced scientists (particularly women) in the South, who now account for about two-thirds of the members in the majority of TDR's scientific committees. The reports from these groups have provided scientific foundation and boosted research efforts for progress in the elimination of diseases such as leprosy, onchocerciasis, schistosomiasis, Chagas disease, lymphatic filariasis, and visceral leishmaniasis.

By 2008, TDR adapted and built on this approach to create groups that systematically reviewed research evidence and provided independent advice and guidance on priority areas and critical research gaps and needs that could be addressed by TDR and by the scientific community at large. Their work resulted in the first-ever WHO Technical Report Series issues dedicated to research priorities on infectious diseases of poverty and offered the basis for the Global Report for research on infectious diseases of poverty published in April 2012 [7]–[11]. As a *Lancet* editorial described it: “TDR focuses on the need for a holistic approach to research that links environment, climate, social factors, and animal health with human health. It calls for attention to health systems, innovation, and technology to fight the diseases of poverty” [12]. The current TDR strategy (2012–2017) is informed by this work and seeks to follow an integrated approach to implementation research to support health systems and disease control activities [13].

## Cutting Edge Research in Support of Disease Control

As part of the United Nations system and WHO, TDR is seen as a neutral platform for stakeholders to discuss, harmonize their activities, and collaborate. This convening power, combined with the scientific credibility of the staff and advisors, has allowed the Programme to introduce cutting-edge themes on the global research agenda for the control of neglected tropical diseases. By pioneering new lines of research, the programme has provided support to specialized training and North–South partnerships, strengthening the capacity and engagement of disease-endemic country (DEC) scientists. One example is the outcome of high-level consultation and planning meetings in 1991 that established a 20-year research plan for the genetic modification of the *Anopheles gambiae* mosquito to render it incapable of harbouring or transmitting Plasmodium parasites [14]. In the following decades, numerous collaborators in 19 countries worked to identify parasite-inhibiting genes, genetically modify mosquitoes, and drive the selection of genes in natural populations. Molecular entomology advanced globally as a discipline, and many African scientists were part of this process.

Examples include genome sequencing of the parasites that are responsible for leishmaniasis, schistosomiasis, sleeping sickness, and Chagas disease, the trypanosomatid sequences published in 2005; the consortium convened by TDR in 1999 for the sequencing of the *Anopheles gambiae* genome, which completed the work and published in 2002; the *Schistosoma mansoni* network for DNA analysis, the first sequenced flatworm with results published in 2009; and another initiative established in 2004 with seed funding from TDR, the Wellcome Trust, NIH, and the Burroughs Wellcome Fund, setting out to map the genome sequence of the Tsetse fly (*Glossina morsitans*), which was published in 2014 [15]–[18].

In all these cases, with a modest financial investment, TDR could trigger and leverage far greater financial and research investments by promoting the development of human and infrastructure resources, strategies and collaborations. The pay-off in terms of knowledge acquired, capacity building, and engagement of DEC scientists was large. For example, *H3ABioNet: A Sustainable African Bioinformatics Network for H3Africa* grew out of this work [19]. As these advances paved the way for field trials, TDR initiated discussions on the ethical, legal, and social implications of testing and evaluating transgenic mosquitoes [20]. Other networks, platforms, and innovative approaches, including on social determinants of health are described in other articles of this series.

## Bringing a Community Perspective into Research and Implementation

TDR highlighted the importance of socioeconomic research for the control of tropical diseases already in the early nineties. At first, only the relationship between tropical diseases and women was studied, but the focus soon shifted to the broader concept of gender that included issues relating to both men and women. This research area is the topic of one article in this special issue (see Sommerfeld et al.), but suffice it to say that for the last 25 years TDR has constantly advocated for social research to be more than an accessory to field research. Community-based approaches for disease control were developed, validated, and adopted by a number of endemic countries [21].

## Defining Priorities at Country Level

TDR's experience in jointly developing research priorities and conducting studies with endemic communities are good examples of how a participatory and locally defined research agenda can produce public health impact. Two examples are summarized here:

### Solving bottlenecks for onchocerciasis control in Africa

TDR had an undisputed success in the development of onchocerciasis control strategies. Developed together with the African Program for Onchocerciasis Control (APOC) and local scientists, evidence on how ivermectin distribution led by communities was more effective than past measures led to a game-changing strategy. The system is now a model of community-based care that is providing treatment to 98 million people in 24 sub-Saharan African countries and has also been found to increase malaria diagnosis and treatment [22]–[23].

### Developing tools and redesigning strategies for visceral leishmaniasis control

The agreement to eliminate visceral leishmaniasis in the Indian subcontinent by 2015 was signed by the ministers of health of Bangladesh, India, and Nepal. Since then, TDR has brought together high-level representatives of the countries to identify research needs and develop both shared and individual strategies going forward (http://www.who.int/tdr/news/2012/vl_elimination/en/). Some of the resulting research has been funded by TDR, but much more has come from collaborations that bring together country public health officials with WHO's Department of Neglected Tropical Diseases, the Drugs for Neglected Diseases initiative (DNDi), One World Health (OWH), and Grand Challenge Canada. They have collectively developed novel approaches for controlling the sandfly vectors that transmit the disease, and an effective strategy for point-of-care diagnosis and treatment close to endemic villages. This research has helped the countries to adapt policies and practices as well as to reduce visceral leishmaniasis incidence [24].

These projects have shown a faster uptake of research results by policy-makers and by the community as a whole when the endemic country stakeholders are part of the process of defining priorities and participating in the studies.

## Performance Assessment Framework to Track Effectiveness

In consultation with key stakeholders, TDR has developed a set of performance indicators to track progress against specifics goals. The framework has evolved to adjust to strategic changes and it is now a core tool behind the principles of equitable research agenda setting. It emphasizes enhanced ownership and utilization of research by disease-endemic countries, and includes values such as equity, inclusiveness, transparency, credibility, and practicality. A performance report is published annually. It measures the extent to which equity issues, such as gender balance and other social determinants of health, are mainstreamed in the portfolio and the extent to which disease-endemic countries have an influential/critical/leadership participation in TDR research-related activities, from research priority setting and partnerships to strengthening policy-making. This framework not only supports TDR but has inspired other organizations to measure research results in a comprehensive way.

## Recent Global Developments

With emerging threats from new infectious diseases and resistance growing to the current drugs, WHO Member States have called for the establishment of a global mechanism to monitor health R&D and further innovations in how R&D can be supported. As cracks are beginning to show in market-based approaches, setting the right research priorities is perhaps more important today than it has ever been. The World Health Assembly called for the establishment of a global observatory for health R&D [25], which would draw together online data on the funding for research. For example, G-FINDER annual surveys [26] could be combined with other metrics such as bibliometric output, patent data, progress in the product pipeline, products licensed, and new evidence-based policy adopted [27].

The challenge is large. Only 37% of countries report health R&D data, but the potential is huge through the opportunity provided by the low cost of sharing data via the Internet. Such a global map would enable better, evidence-informed analysis of priorities at a global level. The World Health Assembly has requested that TDR, with its Member State–driven governance arrangements, work on determining how these priorities could be turned into new calls for research that continue to have the needs of developing countries at their heart [28].
